# Structural and dynamic insights into the role of conformational switching in the nuclease activity of the *Xanthomonas albilineans* Cas2 in CRISPR-mediated adaptive immunity

**DOI:** 10.1063/1.4984052

**Published:** 2017-05-19

**Authors:** Donghyun Ka, Suji Hong, Ugeene Jeong, Migyeong Jeong, Nayoung Suh, Jeong-Yong Suh, Euiyoung Bae

**Affiliations:** 1 Department of Agricultural Biotechnology, Seoul National University, Seoul 08826, South Korea; 2 Department of Pharmaceutical Engineering, Soon Chun Hyang University, Asan 31538, South Korea; 3 Research Institute of Agriculture and Life Sciences, Seoul National University, Seoul 08826, South Korea; 4 Center for Food and Bioconvergence, Seoul National University, Seoul 08826, South Korea

## Abstract

Clustered regularly interspaced short palindromic repeats (CRISPRs) and CRISPR-associated (Cas) proteins constitute a microbial, adaptive immune system countering invading nucleic acids. Cas2 is a universal Cas protein found in all types of CRISPR-Cas systems, and its role is implicated in new spacer acquisition into CRISPR loci. In subtype I-C CRISPR-Cas systems, Cas2 proteins are metal-dependent double-stranded DNA (dsDNA) nucleases, and a pH-dependent conformational transition has been proposed as a prerequisite for catalytic action. Here, we report the crystal structure of *Xanthomonas albilineans* Cas2 (XaCas2) and provide experimental evidence of a pH-dependent conformational change during functional activation. XaCas2 crystallized at an acidic pH represented a catalytically inactive conformational state in which two Asp8 residues were too far apart to coordinate a single catalytic metal ion. Consistently, XaCas2 exhibited dsDNA nuclease activity only under neutral and basic conditions. Despite the overall structural similarity of the two protomers, significant conformational heterogeneity was evident in the putative hinge regions, suggesting that XaCas2 engages in hinge-bending conformational switching. The presence of a Trp residue in the hinge region enabled the investigation of hinge dynamics by fluorescence spectroscopy. The pH dependence of the fluorescence intensity overlapped precisely with that of nuclease activity. Mutational analyses further suggested that conformational activation proceeded via a rigid-body hinge-bending motion as both D8E and hinge mutations significantly reduced nuclease activity. Together, our results reveal strong correlations between the conformational states, catalytic activity, and hinge dynamics of XaCas2, and provide structural and dynamic insights into the conformational activation of the nuclease function of Cas2.

## INTRODUCTION

Clustered regularly interspaced short palindromic repeats (CRISPRs) are a class of repetitive genetic elements discovered in microbial genomes.^1–5^ They consist of short invariably repeat sequences interspaced with similarly sized variable spacer sequences derived from foreign nucleic acids.^6–8^ CRISPR-associated (*cas*) genes lie adjacent to the CRISPR loci and encode a series of conserved proteins exhibiting nucleic acid-related functions.^9–11^ The CRISPR array and the Cas proteins constitute an adaptive immune system in bacteria and archaea, countering invasion by phages and plasmids.^12–15^ During the adaptation stage of CRISPR-mediated adaptive immunity, new spacers are acquired from foreign nucleic acids and integrated into the CRISPR loci of the host genome. Next, the CRISPR loci are transcribed and processed to generate small mature CRISPR RNAs (crRNAs), which assemble with Cas protein(s) to form RNA-protein complexes. Finally, these effector complexes recognize and degrade re-invading nucleic acids using the crRNAs as guide sequences.

CRISPR-Cas systems can be classified into six types and further into ∼20 subtypes.^15–18^ Cas2 is a universal Cas protein found in all types of CRISPR-Cas systems,^15–17,19^ and plays a role in new spacer acquisition during the adaptation stage of CRISPR-Cas immunity.^20–24^ Cas2 proteins of the various CRISPR subtypes have been characterized both structurally and functionally.^19,25–29^ They share common structural features including a homodimeric state, a two-domain protomer architecture consisting of an N-terminal ferredoxin fold and a C-terminal β-strand segment, and conservation of a pair of catalytic Asp or Glu residues. Several Cas2 homologues exhibit metal-dependent nuclease activities, but with different substrate specificities.^25,27,28^ *Sulfolobus solfataricus* Cas2 cleaved single-stranded RNAs (ssRNAs) with a preference for uracil-rich sequences.^25^ Cas2 proteins from *Bacillus halodurans* (BhCas2), *Streptococcus pyogenes* (SpCas2), and *Xanthomonas oryzae* exhibited non-specific double-stranded DNA (dsDNA) nuclease activities.^27,28^ Interestingly, *Thermus thermophilus* Cas2 was active on both dsDNA and ssRNA substrates.^27^ However, the Cas2 proteins of *Desulfovibrio vulgaris* (DvCas2) and *Thermococcus onnurineus* did not exhibit nuclease functions.^19,26^

It has been shown in various systems that Cas2 interacts with Cas1 to form a stable heterohexameric complex, in which a central Cas2 dimer connects two Cas1 dimers.^22,24,30^ The recent structural and mechanistic studies, much of which are about the subtype I-E CRISPR-Cas system from *Escherichia coli*, revealed that the Cas1-Cas2 complex plays crucial roles in several processes of the CRISPR adaptation, including the foreign DNA capture, the recognition of the CRISPR locus, and the integration of new spacers into the CRISPR array.^20,21,31^ The Cas1 proteins in the complex show affinity for DNA segments complementary to the specific target recognition motif, called a protospacer-adjacent motif.^32^ Upon the DNA binding, the Cas1-Cas2 complex undergoes a significant conformational change.^32,33^ In several systems, the Cas1-Cas2 adaptation complexes are capable of independently recognizing the integration sites within the CRISPR loci based on their intrinsic affinities,^23,24^ whereas the integration host factor, a host protein assisting the recognition of the CRISPR array, was discovered in *E. coli*.^34,35^ The Cas1-Cas2 complex also functions as a “molecular ruler” to control the length of spacers,^32,33^ and generates free 3′-OH ends of the bound DNA substrate required for the nucleophilic attacks to the CRISPR array for the spacer insertion.^23,32,36^

In the subtype I-C CRISPR-Cas systems, the Cas2 proteins are metal-dependent dsDNA nucleases, the activities of which are strongly influenced by pH.^27,28^ Previous studies on BhCas2 and SpCas2 found that dsDNA nuclease activities fell significantly at acidic pHs and proteins crystallized under acidic conditions appeared to adopt catalytically inactive conformations.^27,28^ In the crystal structures, the side chains of the two catalytic Asp residues were located too far apart to allow them to coordinate a single catalytic metal ion. The Asp residues of DvCas2, another subtype I-C homologue, were also remotely located.^26^ Based on these observations and other experimental data, it was previously proposed that to become catalytically competent, Cas2 proteins may require a pH-dependent conformational change reducing the distance between the two Asp residues to that required for effective binding of the catalytic metal ion.^27,28^

In this study, we describe the structural and functional characterization of *Xanthomonas albilineans* Cas2 (XaCas2) of a subtype I-C CRISPR-Cas system and provide experimental evidence of pH-dependent conformational switching involving a hinge-bending motion during activation of the dsDNA nuclease function. The crystal structure of the XaCas2 dimer exhibited structural heterogeneity in the putative hinge regions between the two protomers. The presence of a Trp residue in the hinge region allowed us to study the hinge-bending conformational transition with the aid of fluorescence spectroscopy. The pH dependence of the fluorescence intensity overlapped precisely with that of nuclease activity, suggesting that hinge-bending dynamics and catalysis were directly connected. Mutational analysis of XaCas2 further confirmed that conformational activation was in play. Thus, our results provide structural and dynamic insights into the pH-dependent functional activation of XaCas2 and reveal strong connections between structure, function, and dynamics.

## RESULTS

### The crystal structure of XaCas2 adopted a catalytically inactive conformational state

The crystal structures of XaCas2 were determined to resolutions of 1.65 Å and 1.75 Å at 20 and 4 °C, respectively. Data collection and refinement statistics are summarized in Table I. The root mean square deviation (RMSD) of the Cα atomic positions between the two XaCas2 structures was only 0.07 Å, indicating that the two structures were essentially identical. We hereafter describe only the 20 °C structure, which was determined at a higher resolution. The asymmetric unit contained two XaCas2 protomers forming a single dimer with pseudo-two-fold symmetry, 175 water molecules, and three acetate ions. Size-exclusion chromatography revealed that XaCas2 was also in a dimeric state in solution (Fig. S1 of supplementary material). Several residues in the flexible loop region (residues 12–13 in protomer B), the C-terminal tails (residues 87–96 in protomer A and 86–96 in protomer B) and the (His)_6_ tags were not modeled in the final structure due to insufficient electron density.

**TABLE I.** Data collection and refinement statistics.^a^

	20 °C	4 °C
Space group	P6_3_	P6_3_
Unit cell parameters (Å)	a=b= 90.4, c= 50.8	a=b =90.6, c= 50.1
Wavelength (Å)	0.9793	0.9793
Data collection statistics		
Resolution range (Å)	50.00–1.65	50.00–1.75
	(1.71–1.65)	(1.81–1.75)
Number of reflections (measured/unique)	345 997/28 581	292 642/23 845
Completeness (%)	99.7 (100.0)	99.9 (100.0)
R_merge_^b^	0.071 (0.809)	0.067 (0.700)
Redundancy	12.1 (11.5)	12.3 (12.3)
Mean I/σ	33.1 (3.1)	35.8 (3.7)
Refinement statistics		
Resolution range (Å)	29.59–1.65	19.66–1.75
R_cryst_^c^/R_free_^d^ (%)	16.5/20.9	15.9/19.5
RMSD bonds (Å)	0.018	0.015
RMSD angles (deg)	1.6	1.5
Average B factor (Å^2^)	39.8	36.3
Number of water molecules	176	174
Ramachandran favored (%)	98.2	96.9
Ramachandran allowed (%)	1.8	3.1

^a^ Values in parentheses are for the highest-resolution shell.

^b^ R_merge_ =Σ_h_Σ|I_i_(h) − ⟨I(h)⟩|/Σ_h_Σ_i_I_i_(h), where I_i_(h) is the intensity of an individual measurement of the reflection and ⟨I(h)⟩ is the mean intensity of the reflection.

^c^ R_cryst_ = Σ_h_ǁF_obs_|−|F_calc_ǁ/Σ_h_|F_obs_|, where F_obs_ and F_calc_ are the observed and calculated structure factor amplitudes, respectively.

^d^ R_free_ was calculated as R_cryst_ using 5% of the randomly selected unique reflections that were omitted from structure refinement.

**FIG. 1.**
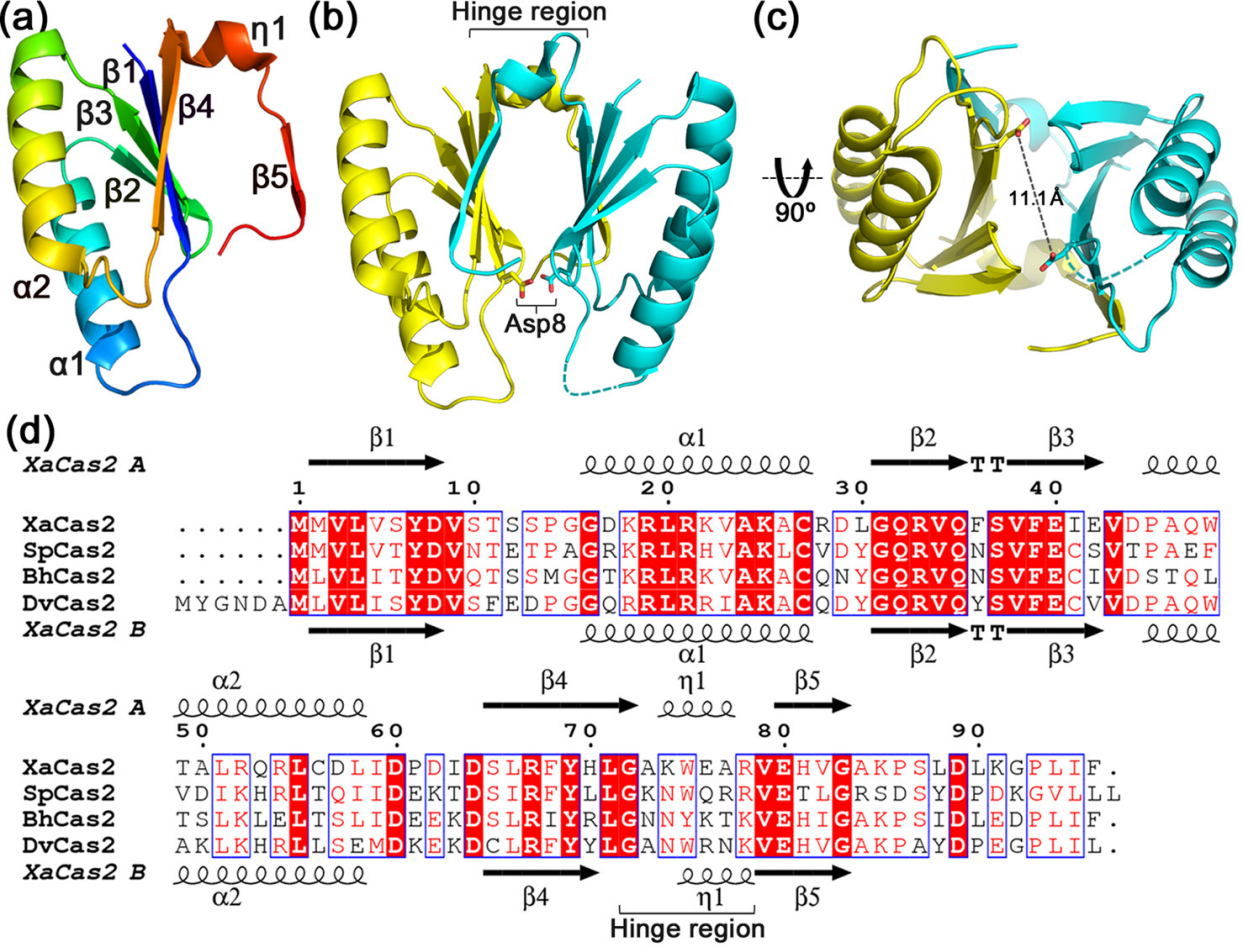
Structure and sequence alignment of XaCas2. (a) Structure of XaCas2 protomer A. XaCas2 protomer includes an N-terminal ferredoxin fold consisting of a four-stranded β-sheet (β1–4) and two α-helices (α1, α2) and a C-terminal segment containing a 3_10_-helix (η1) and a β-strand (β5). (b) Dimeric structure of XaCas2. The two XaCas2 protomers form a single dimer with pseudo-two-fold symmetry. A pair of catalytic Asp8 residues is shown in stick representation. Hinge regions (residues 72–78) are also indicated. XaCas2 protomers A and B are colored in yellow and cyan, respectively. (c) Separation between the two catalytic Asp8 residues in a different view of the XaCas2 dimer structure. The dashed line indicates the distance between the two Asp8 residues. (d) Sequence alignment of Cas2 homologues of subtype I-C CRISPR-Cas systems. Secondary structural elements and hinge regions (residues 72–78) are indicated based on XaCas2.

Overall, the structural features of XaCas2 were similar to those of other subtype I-C Cas2 structures, including the protomer fold, the dimer interface, and the conformational state. The XaCas2 protomer contained an N-terminal ferredoxin fold consisting of a four-stranded antiparallel β-sheet (β1–4) and two α-helices (α1, α2), and a C-terminal segment including a 3_10_-helix (η1) and a β-strand (β5) [Fig. 1(a)]. The C-terminal β5 strand of one protomer was aligned with the β4 strand of the other protomer to extend the four-stranded β-sheet [Fig. 1(b)]. Such β5 swapping created two five-stranded antiparallel β-sheets in a single XaCas2 dimer. Dimerization of XaCas2 buried 1346 Å^2^ of the surface area and involved polar interactions between the two protomers including Gln35-Ser6, Gln35-Ser65, Glu40-Arg67, and Glu40-Tyr69 (Fig. S2 of supplementary material). These interactions are conserved in the other subtype I-C Cas2 structures [Fig. 1(d)].^26–28^

In our crystal structure, XaCas2 seemed to adopt a catalytically inactive conformational state, as was also true of several other subtype I-C Cas2 structures evaluated previously.^26–28^ In the XaCas2 dimer, the two conserved Asp8 residues were too far apart to allow them to coordinate a single catalytic metal ion together [Fig. 1(c)]. The distance between the side chains of the two Asp8 residues was 11.1 Å, and no metal ion was found near the residues. It is thus very likely that XaCas2 must undergo conformational switching to bring the two Asp8 residues sufficiently close to allow them to coordinate the single metal ion required for catalysis. Previous studies on subtype I-C Cas2 proteins revealed that their catalytic functions were strongly pH-dependent as nuclease activities decreased significantly at acidic pHs.^27,28^ These data, and the fact that we crystallized XaCas2 under acidic conditions, suggested that XaCas2 would require a pH-dependent conformational change to become fully active under more basic conditions. We tested this hypothesis by analyzing the nuclease function and structural dynamics of XaCas2.

### XaCas2 is a metal- and pH-dependent dsDNA nuclease

**FIG. 2.**
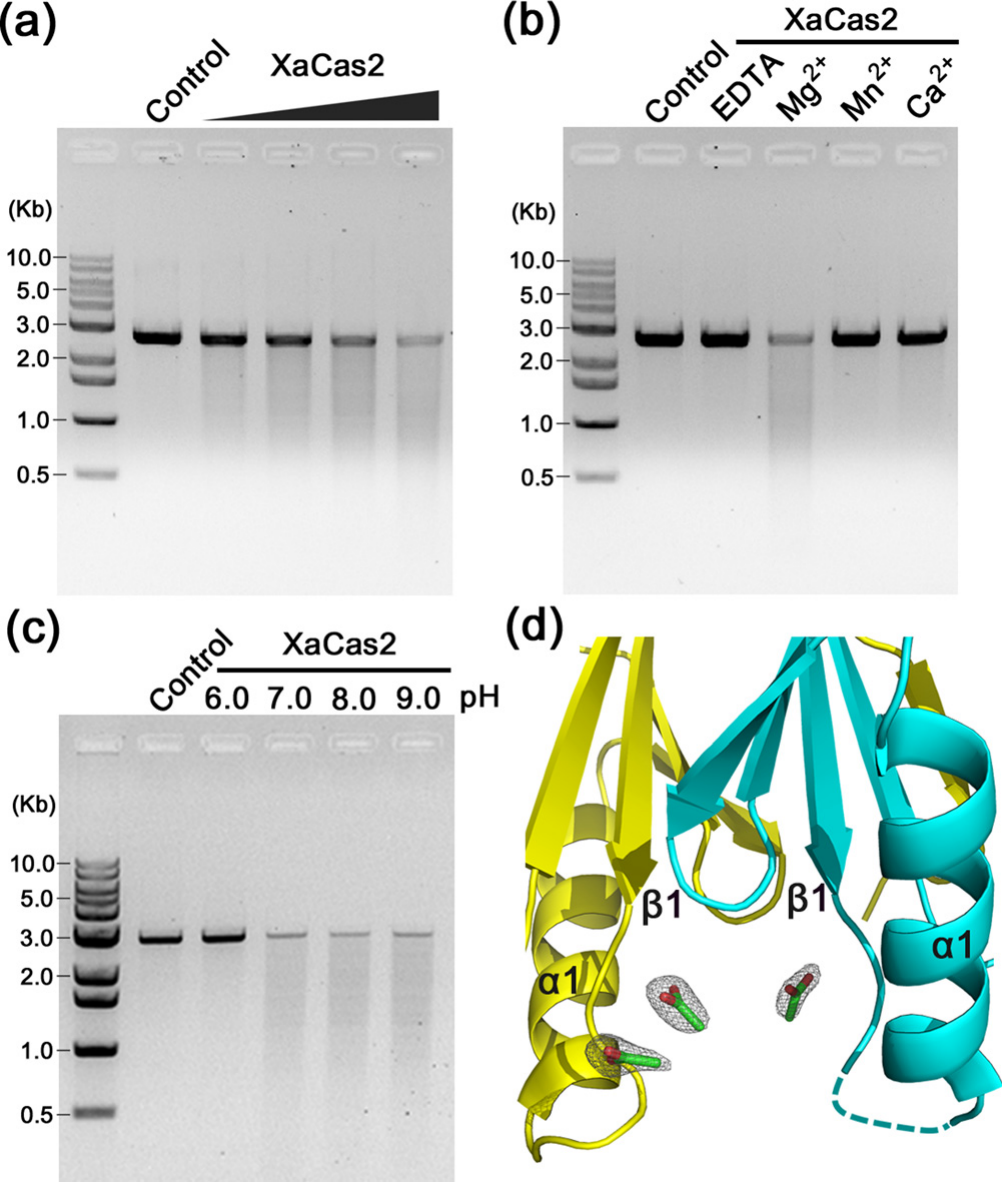
dsDNA nuclease activity of XaCas2. (a) Cleavage of dsDNAs by XaCas2. Linearized pU19 plasmid was incubated with increasing amounts (5, 10, 20, and 40 *μ*M) of XaCas2. (b) Metal dependence of the dsDNA nuclease activity of XaCas2. The dsDNA substrate was incubated with XaCas2 in the presence of divalent ions or EDTA. (c) pH dependence of the dsDNA nuclease activity of XaCas2. The substrate and XaCas2 were incubated in reaction buffer of different pHs. (d) Acetate ions in the proximity of the dsDNA substrate recognition loop. Three acetate ions incorporated during crystallization were identified near the β1–α1 loops of XaCas2 in the asymmetric unit. The acetate ions are shown in stick representations. The 2mF_obs_ - DF_calc_ map is contoured at 1.0 σ for the acetate ions.

Several subtype I-C Cas2 proteins have been reported to contain non-specific dsDNA nuclease functions.^27,28^ XaCas2 exhibited dsDNA nuclease activity when the linearized pUC19 plasmid was used as the substrate. At pH 8.0, XaCas2 cleaved the dsDNA substrate in an XaCas2 concentration-dependent manner [Fig. 2(a)]. This confirmed that the subtype I-C Cas2 homologues shared the dsDNA nuclease function. XaCas2 nuclease activity was metal-dependent as an addition of EDTA abolished DNA cleavage [Fig. 2(b)]. Under our experimental conditions, XaCas2 required Mg^2+^ for activity. Neither Mn^2+^ nor Ca^2+^ was acceptable. In contrast, BhCas2 activity was supported by several different divalent metal ions including Mg^2^, Mn^2+^, Ca^2+^, Ni^2+^, and Fe^2+^.^27^

An earlier study on BhCas2 suggested that the loops connecting β1 and α1 in subtype I-C Cas2 proteins were responsible for recognizing the dsDNA substrates. This was based on a comparison of homologues of other CRISPR subtypes that exhibited different substrate preferences.^27^ The crystal structure of XaCas2 yielded a further piece of evidence supporting the idea that the β1–α1 loop plays a role in substrate recognition. The asymmetric unit of the XaCas2 structure includes three acetate ions incorporated during crystallization. They are found adjacent to the two β1–α1 loops (residues 9–15) of the XaCas2 dimer, and are in close contact with several residues in these regions [Figs. 2(d) and S3 (supplementary material)]. One acetate ion forms hydrogen bonds with Asp8 and Val9 of protomer A, and also seems to be stacked on the side chain of Phe36 of protomer B. Another ion, located close to the first ion, is hydrogen-bonded with Ser10, Thr11, and Ser12 of protomer A. Near the β1–α1 loop of protomer B, we found a third acetate ion, symmetric to the first ion located near protomer A. The absence of another acetate ion near protomer B is probably related to the insufficient electron density of residues 12 and 13 of protomer B.

Based on these observations, and the similarities in molecular structure, we speculate that the acetate ions may mimic the phosphate backbones and/or other regions of the dsDNA substrates, strongly suggesting that residues in the β1–α1 loops are involved in dsDNA substrate recognition by subtype I-C Cas2 proteins. In addition, notably, the crystal structure of the *E. coli* Cas1-Cas2-dsDNA complex showed that residues in the β1–α1 loop of *E. coli* Cas2 were involved in dsDNA binding. When we aligned XaCas2 and the *E. coli* complex by reference to their Cas2 protomers, the acetate ions in the XaCas2 structure are overlapped with the DNA backbone of the *E. coli* complex (Fig. S4 of supplementary material).

When assayed at various pHs, XaCas2 exhibited strong pH-dependent dsDNA nuclease activity [Fig. 2(c)]. Although activity was observed under neutral and basic conditions, no dsDNA cleavage was detected at pH 6.0, consistent with the suggestion that the XaCas2 structure crystallized under acidic conditions was in a catalytically inactive conformation. Together, the functional and structural analyses indicate that XaCas2 is a metal- and pH-dependent non-specific dsDNA nuclease, and support the hypothesis that subtype I-C Cas2 proteins undergo a pH-dependent conformational change to allow metal-binding prior to catalysis.

### XaCas2 protomers exhibited conformational heterogeneity in the putative hinge regions

An earlier study on BhCas2 suggested that the conformational change required for catalytic metal binding involved a rigid-body hinge-bending motion.^27^ Previously, we proposed that the regions connecting the N-terminal ferredoxin folds and the swapped β5 strands in the C-terminal segments might serve as hinges based on structural comparisons of subtype I-C Cas2 homologues.^28^ Structural analyses of XaCas2 identified residues between the β4 and β5 strands as putative hinge regions, suggesting that the hinge-bending motion is a common feature of subtype I-C Cas2 proteins. We used two hinge-prediction programs running different algorithms. HingeProt, which utilizes an elastic network model,^37^ predicted that Ala73 and Glu76 served as hinges within XaCas2. H-Predictor, which performs thermodynamic estimation,^38^ identified Lys74, Trp75, and Val79 as hinge components. All of these residues are within or adjacent to the region connecting the β4 and β5 strands.

**FIG. 3.**
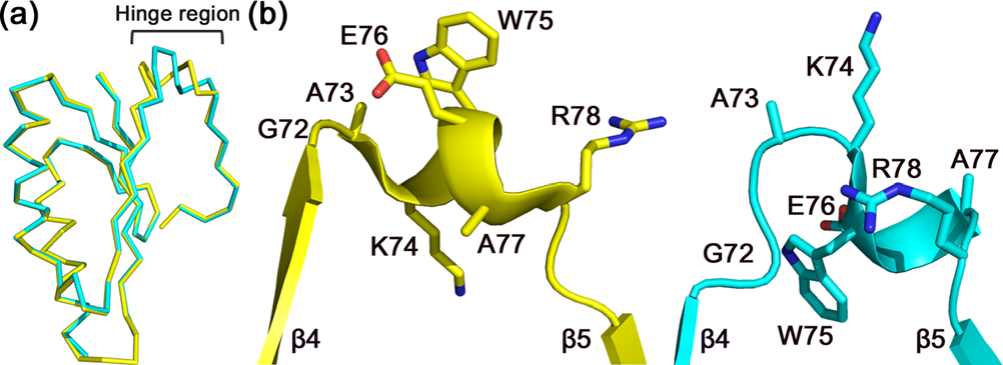
Conformational heterogeneity in the hinge regions of XaCas2. (a) Structural alignment of the two XaCas2 protomers. When XaCas2 protomers A and B, shown in yellow and cyan, respectively, were structurally aligned, a substantial structural deviation was noted in the hinge region (residues 72–78). (b) Side-by-side comparison of the hinge regions of protomer A (left) and protomer B (right). The residues in the hinge regions exhibit completely different side-chain conformations and main-chain displacements between the two protomers.

Strikingly, despite the high similarity in overall structure, the two XaCas2 protomers exhibited significant conformational heterogeneity within the putative hinge regions. When the two XaCas2 protomers were structurally aligned, a substantial structural deviation was noted in residues 72–78 [Fig. 3(a)]. Without these residues, the RMSD of the Cα atomic positions between the two XaCas2 protomers was only 0.3 Å, whereas the RMSD value for residues 72–78 was 3.5 Å. This indicated that structural variation was significant within the putative hinge region only.

Structural variation in the two XaCas2 protomers commences at Gly72, which is conserved in subtype I-C Cas2 proteins [Figs. 1(d) and S5 (supplementary material)]. In protomer A, Gly72 belongs to the β4 strand and the carbonyl oxygen is hydrogen-bonded (2.7 Å) to the backbone nitrogen of Met2 of the β1 strand. However, in protomer B, Gly72 is remote (>4 Å) from the β1 strand and the carbonyl oxygen points toward a different region of the protomer. Consequently, Gly72 of protomer B is not a component of the secondary structural element, and the β4 strand of protomer B is shorter than that of protomer A. Considering that Gly exhibits unique conformational flexibility and that Gly72 is conserved among subtype I-C Cas2 homologues, the Gly residue may play a crucial role as the initiator of hinge-bending. Such conservation also suggests that a conformational change involving a hinge-bending motion is common to subtype I-C Cas2 proteins.

Conformational heterogeneity became more pronounced in the residues that followed Gly72. In particular, Lys74 and Trp75 exhibited completely different side-chain conformations, and main-chain displacements, between the two protomers [Fig. 3(b)]. In protomer A, Lys74 formed salt bridges with Glu40 and interacted hydrophobically with Met2, whereas Trp75 was largely exposed to the solvent. In contrast, the side chain of Lys74 was fully exposed in protomer B, and Trp75 was in close hydrophobic contact with other residues such as Met2 and Leu71. The accessible surface area of Trp75 was 191 Å^2^ in protomer A, but only 41 Å^2^ in protomer B. The conformational heterogeneity persisted to Val79, after which the β5 strand commenced and the two protomers again exhibited high-level structural similarity.

Notably, the putative hinge regions were well defined in the electron-density map, and the B factors were relatively low. The isosurfaces continuously covered not only the main-chain atoms, but also all side-chain atoms in the region except the three terminal atoms (CD, CE, and NZ) of Lys74 of protomer B (Fig. S6 of supplementary material). The average B factor for the main-chain atoms of the hinge regions was lower than that of the entire XaCas2 structure (Table S1 of supplementary material). This suggests that the observed structural heterogeneity is attributable to the presence of two distinct stable structural states rather than a large ensemble of spatially and/or temporally distributed conformations.

### The pH-dependent dynamics of XaCas2 was consistent with the structural and functional analyses

The structural heterogeneity observed in the putative hinge regions is probably caused by differences in crystal contacts within the lattice. However, it is plausible to assume that the two distinct conformations are physiologically relevant, and may represent two different hinge structures corresponding to active and inactive conformational states of XaCas2 at different pHs.

**FIG. 4.**
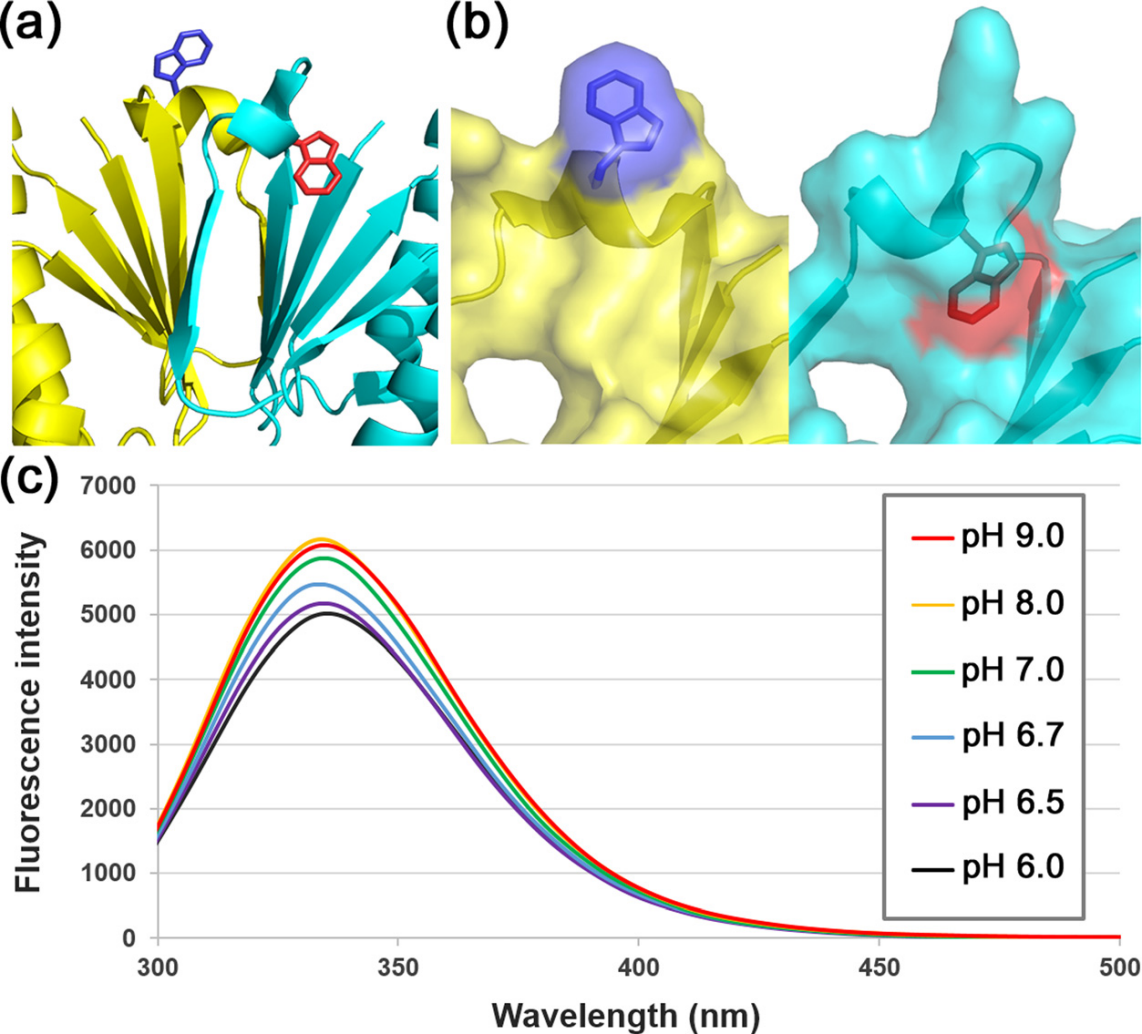
Fluorescence spectroscopy of XaCas2. (a) Different local environments were evident around the Trp75 residue of the hinge regions of the two XaCas2 protomers. XaCas2 protomers are colored as in Fig. 1. Side chains of the Trp75 residues in protomers A and B are shown in blue and red, respectively. (b) Side-by-side comparison of the Trp75 residues of the two XaCas2 protomers in surface representations. Trp75 is exposed to the solvent in protomer A (left), but in close hydrophobic contact with other residues in protomer B (right). (c) Fluorescence emission spectra of XaCas2 at different pHs. The fluorescence intensity of XaCas2 decreased sharply below pH 7.0.

For testing this hypothesis, it was necessary to explore whether the hinge regions adopted different conformations depending on the pH. Fortunately, Trp75 was located in the middle of the hinge region. Trp is a dominant source of protein fluorescence,^39^ and its fluorescence is strongly affected by the microenvironment.^39,40^ Thus, Trp fluorescence can be used to monitor conformational transitions in proteins if Trp residue(s) experience local environmental changes in different conformational states. In the crystal structure of XaCas2, the microenvironment of Trp75 differs dramatically between the two protomers [Figs. 4(a) and 4(b)]. XaCas2 contains another Trp residue, Trp48, but the local environment thereof may not be significantly altered by the proposed conformational change. Trp48 is well buried within the hydrophobic interior of the N-terminal ferredoxin folds of both protomers and probably moves as part of a rigid body during conformational switching of XaCas2.

We thus measured the fluorescence emission spectra of XaCas2 at different pHs [Fig. 4(c)]. XaCas2 yielded almost identical spectra under basic (pHs 8.0 and 9.0) and neutral (pH 7.0) conditions. However, the fluorescence intensity decreased considerably under acidic conditions (below pH 7.0). At pH 6.0, the maximum fluorescence intensity was reduced by ∼20% compared to those at the higher pHs, strongly suggesting that a pH change may considerably alter the local environment around Trp75, reflecting a pH-dependent structural change in the putative hinge region with a concomitant conformational switching of XaCas2.

The change in fluorescence intensity was evident principally over a relatively narrow pH range [6.5–7.0; Fig. 4(c)]. The spectrum at pH 6.5 was similar to that at pH 6.0, whereas the maximum fluorescence intensity at pH 6.7 lay between those at pHs 6.5 and 7.0. This pH range overlapped exactly with that over which XaCas2 exhibits functional switching. In activity assays, XaCas2 displayed robust dsDNA nuclease activities at pHs 7.0, 8.0, and 9.0, but none at pH 6.0 [Fig. 2(c)], indicating that catalytic activity decreased sharply below pH 7.0. The correlation between the pH dependence of fluorescence intensity and nuclease activity suggests that a structural change in XaCas2 is directly related to catalytic activation.

### Mutational analysis of XaCas2 confirmed that conformational switching is required for catalytic activation

**FIG. 5.**
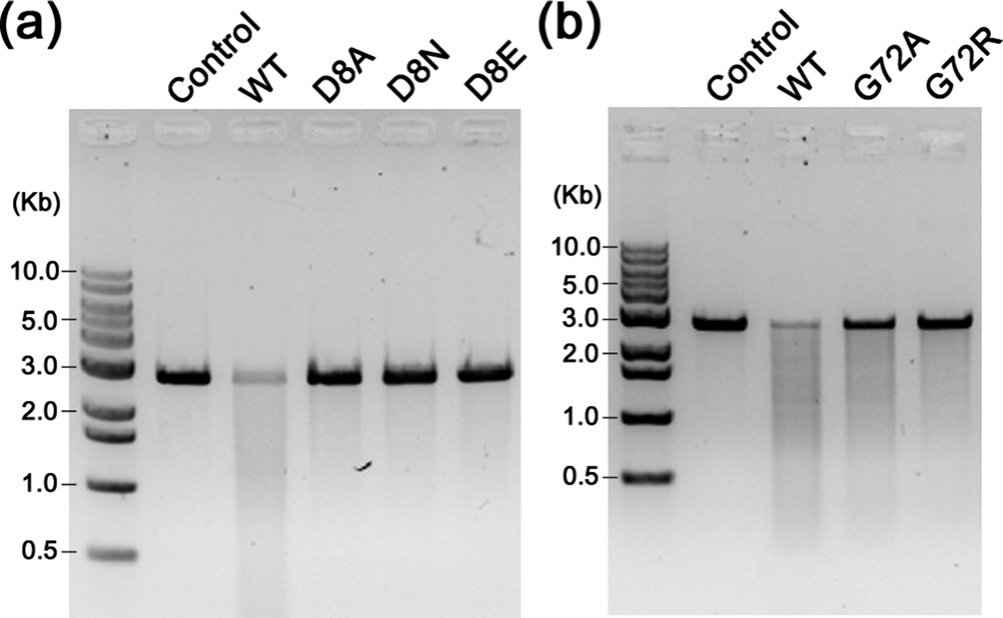
dsDNA nuclease activities of XaCas2 mutants. Cleavage of a dsDNA substrate by XaCas2 mutants containing substituted residues at (a) the catalytic active site and (b) the hinge region. The nuclease activity of XaCas2 was significantly reduced by the mutations of Asp8 and Gly72.

To confirm the direct connection between a conformational change and nuclease function in XaCas2, we constructed several XaCas2 mutants and measured their dsDNA nuclease activities. The circular dichroism spectra of the mutant proteins were similar to that of the wild-type (WT) XaCas2 (Fig. S7 of supplementary material), suggesting the proper folding of the mutants. First, we mutated the conserved Asp8 residue, which supposedly coordinates a metal ion required for catalysis [Fig. 5(a)]. When we mutated the Asp residue to Ala or Asn, cleavage was barely detectable. In addition, no activity was observed when Asp8 was replaced with Glu. This was somewhat surprising since the Glu8 residue of this D8E mutant obviously contained a carboxylic acid group that could be used to coordinate a metal ion. Hence, the presence of a carboxyl side chain alone may not be sufficient to ensure full catalytic activity of XaCas2. Rather, it is likely that the distance between the two carboxylic acid groups is critical to ensure catalytically competent 2:1 stoichiometric binding between the carboxyl side chains and a catalytic metal ion. This strongly suggests that, during catalysis, XaCas2 adopts a conformational state distinct from that observed in the crystal structure, supporting the idea that the conformational transition involves a rigid-body hinge-bending motion.

Next, we constructed XaCas2 mutants in which a residue in the putative hinge region was mutated [Fig. 5(b)]. We mutated Gly72 because this residue is conserved among subtype I-C Cas2 homologues and seemed to be involved in the conformational flexibility evident in the crystal structure of XaCas2. When Gly72 was mutated to Ala, catalytic activity decreased considerably, although the mutation was remote from the catalytic active site (Fig. S8 of supplementary material). Such reduction in nuclease function was more pronounced in a G72R mutant containing a larger side chain, suggesting that conformational flexibility at residue 72 is crucial for the catalytic activity in XaCas2. These results indicated that the hinge region played a critical role in catalytic function, presumably by mediating the structural transition of XaCas2, which is most likely achieved by conformational switching involving a hinge-bending motion.

## DISCUSSION

In this study, we have experimentally confirmed that XaCas2 undergoes a pH-dependent structural change. In previous studies on other subtype I-C Cas2 homologues, pH-dependent conformational switching involving a rigid-body hinge-bending motion was proposed to be a prerequisite for catalytic function based on collective analyses of static structures and functional data.^27,28^ However, direct experimental evidence of a pH-dependent structural change in Cas2 was difficult to find. In the present study, we were able to directly observe a pH-dependent change in the fluorescence intensity of XaCas2, most probably attributable to a pH-dependent structural transition involving the Trp residues of the hinge regions. Moreover, the observed pH dependence in fluorescence correlated precisely with that of catalytic activity measured in functional assays. These findings strongly suggest that the structure, function, and dynamics of the Cas2 protein are interconnected.

Surprisingly, significant conformational heterogeneity was evident within the putative hinge regions between the two XaCas2 protomers. Such variability was previously recognized when Cas2 protomers from different crystal structures were compared,^27,28^ but not between two protomers of a single Cas2 dimer. As the electron densities were well defined and the B factors were relatively low, the two distinct hinge structures of XaCas2 probably represent two stable conformational states, which can be alternatively adopted via movement of the hinge region residues. Hence, it is conceivable that the heterogeneity is physiologically relevant rather than a crystallographic artifact, and that the two distinct hinge structures represent those of the catalytically active and inactive conformational states of XaCas2.

In the XaCas2 structure, Trp75 of the hinge region is exposed to the solvent in protomer A but is buried by the hydrophobic residues in protomer B. In our pH-dependence experiments, both the fluorescence intensity and the nuclease activity of XaCas2 decreased sharply below pH 7.0. As Trp is more fluorescent in a hydrophobic environment,^39,40^ we propose that the hinge region of protomer A with the exposed Trp residue may represent the structural state of catalytically inactive XaCas2 under acidic conditions. On the other hand, the hinge region of protomer B, in which Trp75 is involved in hydrophobic contacts, presumably resembles the structural state at basic pHs, corresponding to the catalytically competent conformational state of XaCas2.

All known subtype I-C Cas2 crystal structures, including that of XaCas2, determined in the present study, adopt catalytically inactive conformational states in which the two conserved Asp residues are remotely located.^26–28^ This conformation may simply be more readily packed in a crystal lattice. It is also possible that the catalytically competent conformational state is substantially populated only after dsDNA molecules are bound. In the absence of substrate, the inactive conformation evident in the crystal structures may be more stable. Indeed, we found extensive cross-protomer contacts at the dimerization interface of the crystal structure of XaCas2, several of which are conserved in other subtype I-C Cas2 homologues (Fig. S2 of supplementary material). Consequently, the structural transition commencing from the inactive conformational state must involve disruption of the stabilizing cross-protomer interactions, which may require a dynamic event such as DNA binding.

In the XaCas2 crystal structure, a residue in a putative hinge region interacts closely with conserved interface residues involved in the cross-protomer contacts. Lys74 of protomer A is located close to the conserved salt link formed by Glu40 of protomer A and Arg67 of protomer B and participates in the electrostatic network of the dimerization interface (Fig. S9 of supplementary material). However, no such interaction between the hinge region and the dimerization interface was evident in protomer B, in which the side chain of Lys74 in the hinge region is fully exposed to solvent. This suggests that the structural transition in the hinge region may be coordinated with overall conformational switching mediated by changes in cross-protomer interactions, assuming that the observed conformational heterogeneity in the hinge region is indeed physiologically relevant.

The dsDNA nuclease activity of XaCas2 appears to be well suited to its role in new spacer acquisition during the development of CRISPR-mediated immunity. The Cas2 protein may process foreign nucleic acids into small pieces as spacer precursors or may cleave DNAs at CRISPR loci to allow insertion of new spacers. However, the nuclease activity of XaCas2 seems to be relatively low considering the amount (20 *μ*M) of the enzyme used for the assays. The experimental condition and/or the DNA substrate might not be optimal for the catalytic function of XaCas2. It is also possible that the nuclease activity of Cas2 is intrinsically weak in the subtype I-C CRISPR/Cas systems. In the previous nuclease activity assays for BhCas2 and SpCas2, the enzymes were used in the range of *μ*M concentration levels (20 *μ*M and 64 *μ*M, respectively).^27,28^ In the study of DvCas2, the authors were not able to demonstrate nuclease activity.^26^ It is imaginable that the ancestral protein of the Cas2 proteins might have possessed strong nuclease activity, which has been reduced or lost during evolution to function as a central component of the CRISPR adaptation complex. In the *E. coli* CRISPR-Cas system, the enzymatic activity of Cas2 is not required for the new spacer integration.^22^

The possibility that XaCas2 may serve as an adaptor protein bridging two Cas1 dimers renders the conformational switching of the protein even more intriguing. If XaCas2 plays a central role within the spacer acquisition assemblage as is true of the *E. coli* Cas2 protein, a conformational change in XaCas2 would trigger significant structural rearrangement of the Cas1-Cas2 complex. Both the relative orientation of, and the distance between, the two Cas1 dimers are important in terms of substrate recognition and catalysis of spacer integration. In the *E. coli* CRISPR-Cas system, the Cas1-Cas2 complex undergoes a significant structural transition upon DNA binding, involving a conformational change in Cas2.^32,33^ The conformation of *E. coli* Cas2 in the DNA-bound Cas1-Cas2 complex seems to resemble that of the catalytically inactive state of XaCas2. The two conserved catalytic residues of each *E. coli* Cas2 protomer are further apart in the DNA-bound structure (10.9 Å) than in the unbound Cas1-Cas2 complex (7.4 Å).^22,33^ Hence, the catalytically inactive conformation of XaCas2 may be required to allow the Cas1-Cas2 complex to adopt a structural profile capable of DNA-binding and/or integration during spacer acquisition. This suggests that XaCas2 may play a dual role in CRISPR-Cas immunity, depending on the conformational states. On the one hand, XaCas2 may act as an enzyme exhibiting intrinsic dsDNA nuclease activity, or, on the other, the protein may serve as a central component of the functional spacer acquisition assemblage. Consequently, conformational switching of XaCas2 may be induced not only by a change in pH but also by the formation of the Cas1-Cas2 complex and subsequent DNA binding.

In summary, we offer a much more detailed picture than was hitherto available of conformational activation of subtype I-C Cas2 proteins. A strong correlation was evident among the conformational state, catalytic activity, and hinge dynamics, indicating that the structure, function, and dynamics of XaCas2 are interconnected. In particular, the pH dependence of the dsDNA nuclease activity overlapped precisely with that of Trp fluorescence. The two heterogeneous hinge structures observed in the crystal structure may be physiologically relevant, corresponding to two distinct conformational states of XaCas2. Moreover, the mutational analysis supported the idea that XaCas2 undergoes conformational activation. Together, the data paint a unique picture of an enzyme in which the pH-dependent structural dynamics is closely linked to functional activation and suggest that Cas2 may play a dual role as a dsDNA nuclease or as a flexible adaptor protein within the spacer acquisition assemblage during the development of CRISPR-mediated adaptive immunity.

## MATERIALS AND METHODS

### Cloning, expression, and purification

The synthetic *X. albilineans cas2* gene was cloned into a pET21a vector with a C-terminal (His)_6_ tag. *E. coli* BL21 (DE3) cells transformed with this construct were cultured in LB medium at 37 °C until the optical density at 600 nm reached 0.7. Protein expression was induced by the addition of 0.5 mM isopropyl-β-D-thiogalactopyranoside, followed by incubation at 17 °C for 16 h. Cells were harvested by centrifugation and resuspended in lysis buffer (300 mM NaCl, 5 mM β-mercaptoethanol (BME), 10% (w/v) glycerol, 20 mM MES pH 6.0).

After sonication and centrifugation, the supernatant was loaded onto a 5 mL HisTrap HP column (GE Healthcare, USA) pre-equilibrated with affinity chromatography buffer (300 mM NaCl, 5 mM BME, 10% (w/v) glycerol, 30 mM imidazole, 20 mM MES pH 6.0). After washing the column with the buffer, the bound protein was eluted by applying a linear gradient of imidazole (up to 450 mM). The protein was further purified using a HiLoad 16/60 Superdex75 column (GE Healthcare, USA) equilibrated with size-exclusion chromatography buffer (200 mM NaCl, 2 mM dithiothreitol (DTT), 5% (w/v) glycerol, 20 mM MES pH 6.0).

### Crystallization, data collection, and structure determination

XaCas2 crystals were grown at 20 and 4 °C using the hanging-drop method from 6 mg/mL protein solution in buffer (200 mM NaCl, 5% (w/v) glycerol, 2 mM DTT, 20 mM HEPES pH 7.5) mixed with an equal volume of reservoir solution (23% (w/v) polyethylene glycol 4000, 10% (w/v) glycerol, 85 mM sodium acetate pH 4.6, 50 mM or 100 mM ammonium acetate for growth at 20 and 4 °C, respectively). Crystals were cryoprotected in the reservoir solution supplemented with additional 2.5% (w/v) glycerol and flash-frozen in liquid nitrogen.

Diffraction data were collected at the beamline 7 A of the Pohang Accelerator Laboratory at 100 K. The diffraction images were processed with HKL2000.^41^ The BhCas2 structure (PDB code 4ES2) was used as a starting model for molecular replacement phasing of the XaCas2 structure at 20 °C in PHASER.^42^ A solution for the 4 °C data was found using the 20 °C structure. The final structures were completed using alternate cycles of manual fitting in COOT^43^ and refinement in PHENIX.^44^ The stereochemical quality of the final models was assessed using MolProbity.^45^

### Nuclease activity assay

Linearized pUC19 plasmid (100 ng) and XaCas2 protein (20 *μ*M) were incubated in reaction buffer (100 mM KCl, 2.5 mM MgCl_2_, 20 mM HEPES pH 8.0) at 37 °C for 2 h. When the pH dependence of the activity was tested, increased amounts (200 mM) of the buffer chemicals (MES for pH 6.0, HEPES for pHs 7.0 and 8.0, and CHES for pH 9.0) were added to the reaction buffer. After proteinase K treatment, the reaction products were analyzed on 1% (w/v) agarose gels and visualized using ethidium bromide.

### Fluorescence spectroscopy

Fluorescence spectra were obtained from 1.0 mg/ml solution of XaCas2 in buffer (100 mM KCl, 2.5 mM MgCl_2_, 200 mM HEPES pH 8.0) using a FluoroMate FS-2 fluorescence spectrometer (Sinco, Korea) at room temperature. For measurements at different pHs, MES (pH 6.0), HEPES (pH 7.0), and CHES (pH 9.0) were added to the buffer. Emission data were collected from 300 nm to 500 nm at 1 nm intervals. The excitation wavelength was 282 nm.

### Preparation of XaCas2 mutant proteins

Mutant genes were generated using mismatched PCR primers, and the mutations were confirmed by DNA sequencing. The mutant proteins were expressed and purified as described above for the WT XaCas2.

### Accession numbers

The atomic coordinates and structure factors of XaCas2 at 20 and 4 °C were deposited in the Protein Data Bank^46^ with the accession codes 5H1O and 5H1P, respectively.

## SUPPLEMENTARY MATERIAL

See supplementary material for the further detailed description of the XaCas2 structure (Table S1 and Figures S1–S9).
